# Potential therapeutic effect of NK1R antagonist in diabetic non-healing wound and depression

**DOI:** 10.3389/fendo.2022.1077514

**Published:** 2023-01-04

**Authors:** Mingyu Li, Hao Ma, Shunuo Zhang, Yuan Peng, Liang Ding, Yixin Zhang, Peiru Min

**Affiliations:** ^1^ Department of Plastic and Reconstructive Surgery, Shanghai Ninth People’s Hospital affiliated to Shanghai Jiao Tong University School of Medicine, Shanghai, China; ^2^ State Key Laboratory for Chemistry and Molecular Engineering of Medical Resources, School of Chemistry and Pharmaceutical Sciences, Guangxi Normal University, Guilin, China

**Keywords:** NK1R, aprepitant, T1DM, depression, wound healing

## Abstract

Diabetes is a global disease with huge impacts on patients due to its complications, among which non-healing wounds and depression are common and challenging. The neurokinin 1 receptor (NK1R) inhibitor, aprepitant has been broadly applied for an antidepressant effect in depressive patients. Recent literature has indicated a therapeutic effect of downregulation in NK1R to diabetes-related fracture, cardiomyopathy, gastroparesis, and ocular surface disorders. In this study, differential expression genes in diabetes and depression were analyzed based on several RNA sequencing datasets from the GEO database to confirm NK1R in the overlapping set. Interaction network and gene set enrichment analysis were subsequently conducted. As a result, NK1R-related genes took part in angiogenesis, epithelial-mesenchymal transition (EMT), collagen deposition, and inflammation in diabetes and depression. *In vivo*, the downregulation of NK1R was proved to promote vascular proliferation and enhance diabetic wound healing, which provides a potential therapeutic target for the management of diabetic non-healing wounds and depression.

## Introduction

Diabetes mellitus (DM) is a worldwide health problem associated with severe complications that influence both the patient’s quality of life and survival (1). As one of the major categories, Type 1 DM (T1DM) exhibits hyperglycemia, significant microvascular injury and a chronic inflammatory response thus resulting in peripheral nerve damage and impaired wound healing, especially at sacrococcygeal region and extremities (2, 3). Moreover, patients with T1DM tend to develop psychiatric morbidity (4). It has been stated that more than thirty percent of patients with T1DM may be suffered from depression (5). Although the exact mechanism remains unclear, treatment with insulin cannot simply manage the aforementioned conundrums simultaneously.

Recent studies have demonstrated the key role of neurogenic inflammation (NI) caused by the secretion of the proinflammatory neuropeptides from peripheral neurons in diabetes and depression (6, 7). As the specific receptor of neuropeptide Substance P (SP), the neurokinin-1 receptor (NK1R) is found expressed on neurons, epithelial cells, fibroblasts, and immune cells, thus mediating NI and contributes to inflammatory diseases and psychosomatic disorders (8). Aprepitant, the NK1R antagonist which originally developed for the blockage of the SP-NK1R pathway, has been broadly proved effective and beneficial for varies of depressions by both fundamental researches and clinical trials (9–13). On the other hand, NK1R antagonist demonstrated potential therapeutic effects for diabetic fractures, gastroparesis, and ocular surface diseases (14–16).

Therapeutic targets of diabetes have been widely studied. However, potential effects of aprepitant in diabetic non-healing wound were still lack of investigation. In this study, we hypothesized that NK1R played a vital role through inflammation in the development of T1DM-related non-healing wounds and depression. Moreover, inhibition of NK1R activation and subsequent pro-inflammatory initiation would possess a therapeutic effect in diabetic non-healing wound. The GSE37450 and GSE198597 dataset were used to explore the potential therapeutic effect of aprepitant for T1DM-related non-healing wounds and depression, respectively.

## Materials and methods

### Data acquisition and processing

The RNA sequencing data of T1DM and depression were downloaded from the Gene Expression Omnibus (GEO) database (https://www.ncbi.nlm.nih.gov/geo/). GSE37450 was used to analyze T1DM gene expression and GSE198597 was used to analyze depression gene expression.

### Differential expression analysis

After log2 transformation, we used R (version 4.1.2) to calculate the differential expression of spleen leukocytes between normal and NOD mice in GSE37450. We performed lmFit and eBays functions in the limma package for multiple linear regression and statistical tests to obtain the significance of each differential expressed gene. The same methods were applied to GSE198597 for differential expression of the hypothalamus between normal and depressed C57BL/6N mice.

### Enrichment analysis

We performed Gene set enrichment analysis (GSEA) using the GSEA software (GSEA version: 4.2.3) from Broad Institute (https://www.gsea-msigdb.org/gsea/index.jsp). We downloaded Gene Ontology gene sets containing BP, CC, and MF subsets as well as hallmark gene sets in the Molecular Signatures Database (MSigDB). The minimum number of the gene set was 5, and the maximum number of the gene set was 5000. After a thousand resampling, we obtained the related pathways and molecular mechanisms in MSigDB.

### Correlation and interaction analysis

Protein interaction data were derived from the STRING database (https://string-db.org). The interaction network was depicted in Cytoscape (version 3.4.1). Then, Pearson correlation analysis by the corr. test function of the psych package was performed for the NK1R-related genes in overlapped gene sets. In addition, the heatmaps of overlapped genes in each disease were obtained from a complex heatmap package.

### Animals

C57BL/6 mice (male, 6 weeks old) were supplied by ShengChang Biotech (Shanghai, China). All animal experiments were approved by the Animal Experimentation Ethics Committee of the School of Medicine, Shanghai Jiao Tong University. C57BL/6 mice were accommodated in a controlled habitat and provided with water and rodent food for 1 week to be familiarized with their habitat.

### Diabetes induction

After a week of adaptive feeding, C57BL/6 mice were singly intraperitoneally injected with streptozotocin (STZ, 150mg/kg, Sigma-Aldrich, S0130) in a freshly prepared citrate buffer (0.1 M, pH 4.5). In the next 5 days, the blood glucose was measured by a blood glucose monitor (**Supplementary Materials, Table S1**). The mice that showed blood glucose concentrations above 16.7 mM were considered diabetic and used for further experimentation.

### Full-thickness wound model in diabetic mice

After diabetes induction, a total of 24 diabetic mice were anesthetized by intraperitoneal injection of 10% chloral hydrate (3.5 mL per 1 kg body weight). Before wound fabrication, the dorsal side of the mice was shaved, depilated, and disinfected. Then, an oval wound (about 6 mm in diameter) was created on the dorsum of the mouse. The NK-1R antagonist aprepitant (Med Chem Express, CAS No. 170729-80-3, 15uL/wound) was injected around the wounds immediately. The 24 mice were randomly divided into 4 groups: 0uM (PBS, control) group; 200uM-aprepitant group; 500uM-aprepitant group and 1000uM-aprepitant group. Treatments were performed every day until Day 15. Digital photographs were taken at day 0, 5, 10, and 15. A circular marker (6mm in diameter) was placed around the wound to standardize the measurements. ImageJ software was used to calculate the wound area of each photographic image. Besides, body weight was also established at day 0, 5, 10, and 15.

### Histological analysis

After 15 days, all remaining mice were sacrificed, and the surrounding tissues of wounds were obtained for histological analysis. The wounds were fixed with 4% paraformaldehyde for tissue fixation. After being dehydrated with a series of graded ethanol, the tissues were embedded in paraffin and cut into 5-μm-thick longitudinal section. Then, the sections were stained with hematoxylin and eosin (H&E) and Masson’s trichrome stain. The image was taken by a microscope (Nikon, Japan).

### Immunohistochemistry analysis

The expression of CD31 in wounds was examined using an anti-CD31 antibody (Abcam, ab28364) by a standard immunohistochemistry protocol. Dewaxed sections were incubated with primary anti-CD31 antibody (Abcam, ab28364) at a dilution of 1:300 for 1 h at room temperature and then incubated with goat anti-mouse immunoglobulin (HRP, Abcam, ab6789) secondary antibody for 30 min at room temperature. The image was taken by a microscope (Nikon, Japan).

### Quantitative RT-PCR analysis

After 15 days, all remaining mice were sacrificed, and the surrounding tissues of wounds were obtained for qRT-PCR. Wound skin tissues were stored in liquid nitrogen and total RNA was extracted using TRIzol reagent (Invitrogen, USA) according to the manufacturer’s protocol. Then, 1 µg of total RNA was reverse transcribed into cDNA using the FastKing cDNA synthesis kit (Tiangen Biotech, China). qRT-PCR was performed with TB Green Premix (Takara, Japan). The mRNA expression levels of GAD1, ALDH1B1, FOXP2 were detected. PCR primers were designed based on sequences from the corresponding genes (**Supplementary Materials, Table S2**). All data were normalized using GAPDH as the internal control by the Δ-CT method.

### Western blotting

Total proteins were extracted using RIPA lysis buffer containing phosphatase and protease inhibitors (Beyotime Biotechnology, China). The concentration of total protein was detected with a BCA Protein Assay kit (Beyotime Biotechnology, China). Equal amounts (20 µg) of protein were separated using 4–20% SDS-PAGE gels. The proteins were then transferred to nitrocellulose membranes (0.45µm; Millipore, USA). The membranes were blocked with 5% non-fat milk for 1 h at room temperature and incubated with primary antibodies (**Supplementary Materials, Table S3**) overnight at 4°C. After washing, the membranes were incubated with goat anti-rabbit secondary antibodies (**Table S2**). Finally, the membranes were visualized using the Odyssey CLx Infrared Imaging System (LI-COR Biosciences, Lincoln,Nebraska, USA). GAPDH and β-actin protein intensities were used as internal controls.

### Statistical analysis

Data analysis was performed using GraphPad Prism (9.0.0, America). Continuous variables are presented as the mean ( ± standard deviation, SD). One-way analysis of variance (ANOVA) was performed to compare the means of multiple groups, and if significant, a *post hoc* Tukey’s test was used to explore the pairwise differences between groups. P-values< 0.05 were considered statistically significant.

## Results

### 1 NK1R was both up-regulated in T1DM and depression

**Figure 1:**
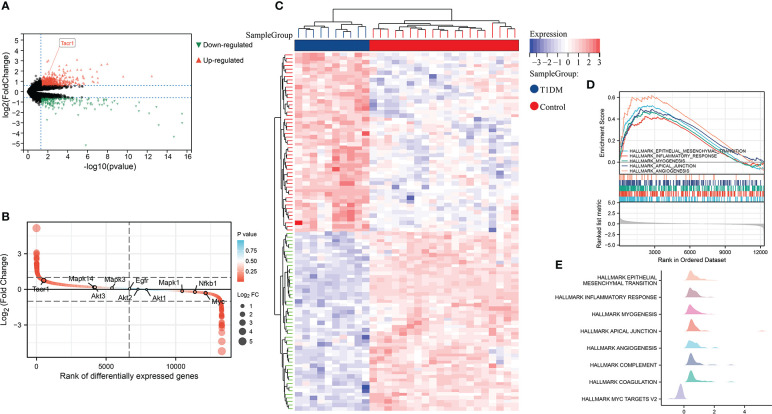
**(A)** Volcano plot of differential expression analysis in GSE37450 dataset. **(B)** Rank of differentially expressed genes in GSE37450 dataset. **(C)**Heatmap of differential expression analysis in GSE37450 dataset. **(D)** Enrichment score and the rank in ordered dataset of GSEA hallmark gene sets in GSE37450 dataset. **(E)** The distribution of gene expression in significant hallmark gene sets in GSE37450. X axis represents log2(Fold Change) of enriched genes.

By differential expression analysis of GSE37450, NK1R was found up-regulated in T1DM. Among all of the differentially expressed genes, NK1R belonged to the top-ranking sets. In hallmark gene sets, we found that differentially expressed genes in T1DM were significantly correlated with epithelial-mesenchymal transition (EMT), inflammatory response, myogenesis, apical junction, and angiogenesis (**Figure 1**).

**Figure 2:**
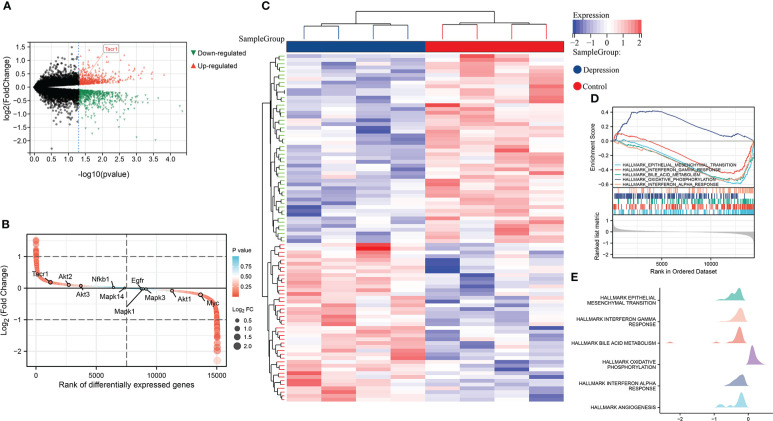
**(A)** Volcano plot of differential expression analysis in GSE198597 dataset. **(B)** Rank of differentially expressed genes in GSE198597 dataset. **(C)**Heatmap of differential expression analysis in GSE198597 dataset. **(D)** Enrichment score and the rank in ordered dataset of GSEA hallmark gene sets in GSE198597 dataset. **(E)** The distribution of gene expression in significant hallmark gene sets in GSE198597. X axis represents log2(Fold Change) of enriched genes.

In GSE198597, we found NK1R was significantly up-regulated in depression and belonged to the top-ranking sets as well. These differently expressed genes in depression were enriched in EMT, interferon response, bile acid metabolism, oxidative phosphorylation and angiogenesis, which was similar to the enrichment analysis of T1DM (**Figure 2**).

### 2 The overlapped genes in T1DM and depression constructed an interaction network

**Figure 3:**
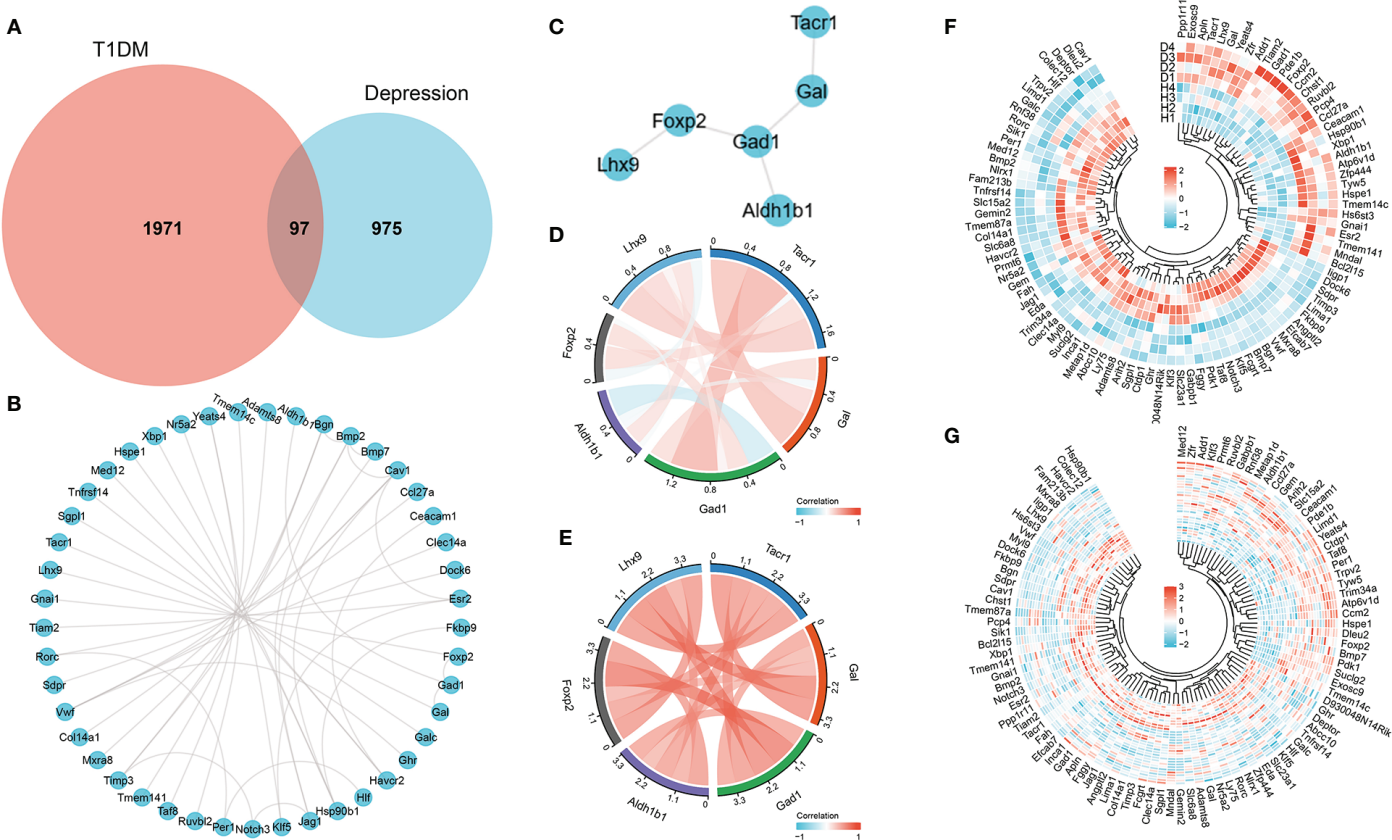
**(A)** Overlapped genes of differential expression analysis in both GSE37450 and GSE198597 dataset. **(B)** Interaction network of overlapped genes. **(C)** Interaction of NK1R-related genes in overlapped gene sets. **(D)** Pearson analysis of NK1R-related genes in overlapped gene sets in GSE37450 dataset. **(E)** Pearson analysis of NK1R-related genes in overlapped gene sets in GSE198597 dataset. **(F)** Heatmap of NK1R-related genes in overlapped gene sets in GSE37450 dataset. **(G)** Heatmap of NK1R-related genes in overlapped gene sets in GSE198597 dataset.

After differential expression analysis of GSE37450 and GSE198597, we found 97 overlapped genes that are differently expressed in both datasets (**Figure 3A**). The interaction network of these overlapped genes from STRING was depicted, and NK1R-related genes (Gal, Gad1, Foxp2, Lhx9, and Aldh1b1) in the network were extracted (**Figures 3B, C**). Then, Pearson analysis was performed to show the correlation among NK1R-related genes (**Figures 3D, E**). In addition, we constructed a heatmap of overlapped genes in each disease to show the expression pattern respectively (**Figures 3F, G**).

### 3 Enrichment analysis of overlapped genes

**Figure 4:**
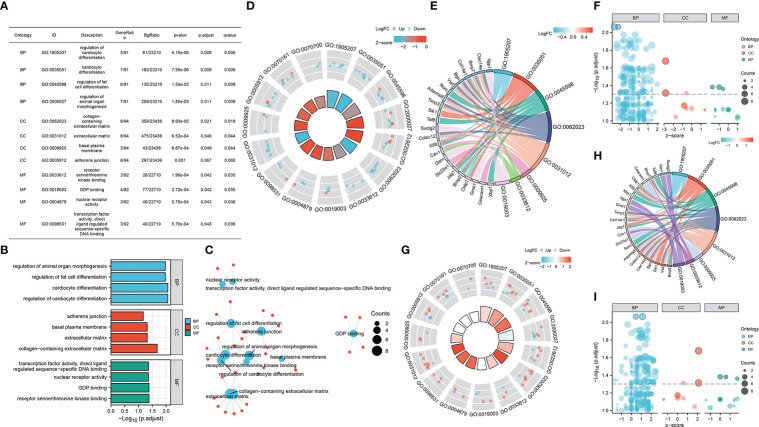
**(A–C)** GO enrichment analysis of overlapped gene sets. **(D–F)** GO enrichment analysis of overlapped gene sets based on GSE37450 expression matrix. **(G–I)** GO enrichment analysis of overlapped gene sets based on GSE198597 expression matrix.

Furtherly, we performed GO enrichment analysis for overlapped genes. In the biological process, these genes were enriched in the regulation of cardiocyte differentiation, fat cell differentiation, and organ morphogenesis. In the cellular component, they were enriched in the adherens junction, basal plasma membrane, and extracellular matrix. As for molecule function, GDP binding, kinase binding, nuclear receptor activity, and transcription factor activity were found enriched by overlapped genes (**Figures 4A–C**). Then, we combined the expression matrix and GO enrichment analysis, constructing the interaction network between overlapped genes and GO terms (**Figures 4D–I**).

### 4 The NK-1R antagonist aprepitant promoted diabetic wound healing *in vivo*

**Figure 5:**
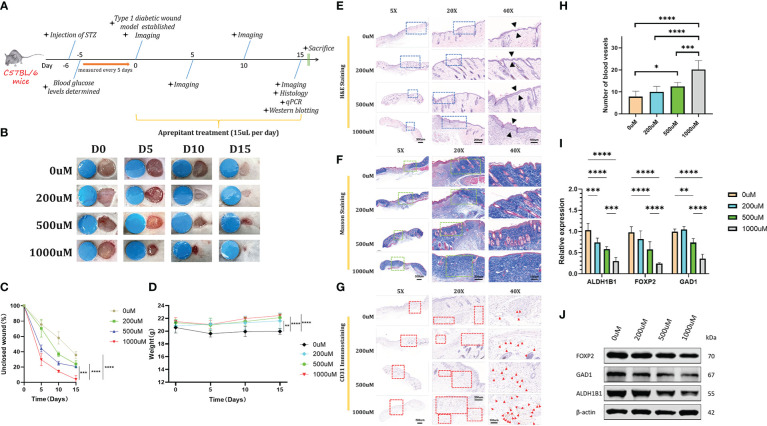
**(A)** Schematic timeline of diabetic wound formation and the following treatments. **(B, C)** Representative images and quantitative measurement of wounds on days 0, 5, 10, and 15 days. **(D)** Changes of body weights of mice after different treatments. **(E, F)** Representative H&E staining images and Masson staining images. **(G, H)** Representative CD31 immunostaining images after different treatments and the corresponding quantitative data of the CD31-positive blood vessels’ numbers from respective staining images (40X). **(I)** qRT-PCR analysis of relative ALDH1B1, FOXP2, and GAD1 levels in different treated groups. **(J)** Expression of ALDH1B1, FOXP2, and GAD1 levels in different treated groups detected by western blotting analysis. Scale bars: 500μm for 5X, 200μm for 20X, and 100μm for 40X. n=6, ns: no significant, *p <.05, **p <.01, ***p <.001, ****p <.0001. Data were presented as mean ± SD. One-way ANOVA with Tukey *post-hoc* test was used.

To explore the potential therapeutic effect of NK-1R antagonist aprepitant for clinical treatment, we established a full-thickness diabetic wound model and used PBS, 200uM aprepitant, 500uM aprepitant, 1000uM aprepitant to treat the diabetic mice (**Figure 5A**). The unclosed wound rate in mice treated with 1000uM aprepitant was the lowest in these different treated groups at days 0, 5, 10, and 15 post-wounding (**Figures 5B, C**). Moreover, the aprepitant-treated groups resulted in the recovery of body weights compared with controled group (**Figure 5D**). Besides, histological analyses were performed to investigate the wound repair efficiency in different treatment groups at day 15 post-operation. H&E staining analysis(5X,20X,40X) showed that 1000uM aprepitant-treated group exhibited the highest re-epithelialization rate among other groups (**Figure 5E**). We also observed the Masson staining analysis which demonstrated that the collagen deposition in the 1000uM aprepitant-treated group was significantly increased when compared to other groups (**Figure 5F**). CD31 is an indicator of blood vessels and very few blood vessels were observed in the wound tissues of the PBS group, while the treatment of 200uM, 500uM, and 1000uM groups improved vascular network formation (**Figure 5G**). Notably, the most CD31-positive blood vessels per field in CD31 immunostaining photographs (40X) was achieved in the 1000uM group (**Figure 5H**). On Day 15, we measured the mRNA level of GAD1, ALDH1B1, and FOXP2 in the wound skins. The results showed that the levels of GAD1, ALDH1B1, and FOXP2 in the 1000uM aprepitant group were reduced compared to the PBS, and 200uM groups (**Figure 5I**). The results of western blotting showed similar concentration-related trend as qRT-PCR (**Figure 5J**). These data indicated that aprepitant significantly accelerated diabetic wound healing by promoting wound re-epithelialization, collagen deposition and angiogenesis, with down-regulated GAD1, ALDH1B1, and FOXP2 expression in wounds.

## Discussion

It is commonly acknowledged that T1DM results from the inflammatory response, exhibiting microvascular injury and damage to peripheral nerves (17). As a critical mediator between neurons and inflammation, NK1R is responsible for the release of proinflammatory cytokines such as CCL4, CXCL2, MCP-1, CCL5, and IL-8, which recruit monocytes, macrophages, and lymphocytes thus leading to the pathogenesis of various inflammatory diseases (18). Neuroimmune interactions with macrophages controlled islet destruction, indicating the control of neuroimmune interactions may represent a new therapeutic target for T1DM (19). Recent reports showed that targeting NK1R was effective in several diabetes-related diseases. Carlin et al. (15) found that the application of NK1R antagonist, and tradipitant clinically improved the patients with diabetic gastroparesis. Jeong et al. (20) described the effects of selective NK1R antagonist RP-67580 in diabetic atria. Although there are studies illustrating the association between NK1R and diabetic complications, e.g., corneal wound healing, fracture, and limb ischemia, evidence-based insight into the exact mechanisms were still lacking (14, 21, 22). As diabetic non-healing wounds and depression are highly correlated with chronic inflammation while NK1R plays a central role in the development of inflammation *via* angiogenesis, we hypothesized NK1R as a potential therapeutic target for both diseases (23).

Based on the results of the bioinformatics analysis, differential gene expression showed that NK1R was significantly upregulated in diabetes and depression, indicating a key role of NK1R in both diseases. According to hallmark gene sets in GSEA, differently expressed genes in diabetes were enriched in EMT, inflammatory response, and angiogenesis, which was also found highly correlated with depression, indicating the potentially shared mechanisms between the two diseases. Then, we performed GO analysis on overlapped genes to clarify the enrichment relationship between diabetes and depression. The results showed that these genes were enriched in organ morphogenesis, fat cell and cardiocyte differentiation, adherens junction, basal plasma membrane, extracellular matrix, GDP and kinase binding, nuclear receptor activity as well as a transcription factor.

To further explore the key proteins related to NK1R which affected the development of diabetes and depression, we conducted PPI analysis and identified the five NK1R-related genes: aldehyde dehydrogenase 1B1 (ALDH1B1), forkhead box P2 (FOXP2), galanin (GAL), glutamate decarboxylase 1 (GAD1) and LIM homeobox 9 (LHX9). Among the five aforementioned genes, GAL has been broadly studied along with the NK1R in neurons and found to interact with life stresses thus mediating depression and anxiety (24–26). Moreover, GAL also regulated insulin release, which was associated with T1DM development (27). ALDH1B1 was found upregulated in the injured pancreas after streptozotocin paradigms and showed an association with diabetes (28, 29). Li T et al. revealed the relationship between FOXP2 and depression (30). As to LHX9, the hypothalamic marker has been proven highly correlated with FOXP2 in patients with depression (30–33). Combined with the enrichment analysis, we provoked the hypothesis that the GAD1, ALDH1B1, and FOXP2 genes functioned with NK1R in terms of angiogenesis, EMT, collagen deposition, and inflammation, while blockade of NK1R may be therapeutic not only for depression but also for the diabetic non-healing wound.

To further verify the hypothesis, C57BL/6 mice were generated as a murine model of a T1DM non-healing wound. Strikingly, we found that topically applied NK1R antagonist, aprepitant could promote the healing of the refractory diabetic wound in a concentration-related manner. qRT-PCR and western blotting were then used for the verification of selected related genes and proteins in the diabetic non-healing wound model. The mRNA level of GAD1, ALDH1B1 and FOXP2 was observed to downregulated in aprepitant-treated mice in a concentration-related and negatively-correlated manner. The expression of these proteins was proved to be downregulated with aprepitant and showed the same concentration-related manner as qRT-PCR. Versus the control group, the thickening epithelial layer and increased collagen deposition could be observed by H&E and Masson staining. CD31 immunohistochemistry showed that a high concentration of aprepitant could potentially increase neovascularization. These phenomenons provided compelling pieces of evidence that aprepitant could effectively promote the healing process of T1DM non-healing wounds through regulation of the aforementioned NK1R-related genes. The underlying mechanism is still controversial. Momen (34) contributed the aprepitant-related angiogenesis to MMP-2, MMP-9, VEGF, and VEGFR. On the other hand, regulation of EGFR, Akt, and SIRT1 by NK1R blockade was also considered beneficial in terms of re-epithelialization and collagen deposition (35–37).

To the best of our knowledge, our study was the first to utilize bioinformatics methods in detecting potential therapeutic targets in diabetic non-healing wound and depression. We explored the common mechanisms of the two diseases, analyzed roles of NK1R-related genes and verified the therapeutic effect of aprepitant. Although the related pathways and key molecules remain not entirely clear, our work provided promising evidences that blockage of the SP-NK1R pathway will downregulate the expression of ALDH1B1, FOXP2 and GAD1, which may be therapeutic for both diseases.

Besides, the neuroinflammation network plays an important role in both diseases, while NK1R could initiate neurogenic inflammation thus leading to neuronal damage (38–40). The release of inflammatory cytokines and the activation of CD8+ T cells contribute to pancreas injury, followed by vasodilation and increased permeability of the microvasculature caused by NK1R (2, 18). Consequently, rescuing the disrupted vascular endothelial cells and relieving neurogenic inflammation through the NK1R pathway stands as a promising therapeutic strategy. Our study also has some limitations. Due to lacking in feasible animal models with both diseases, further *in vivo* study for depression in T1DM mice is yet to be verified. Moreover, in-depth researches need to be carried out to reveal the underlying mechanisms and complex interplay between the two diseases.

## Data availability statement

The datasets presented in this study can be found in online repositories. The names of the repository/repositories and accession number(s) can be found in the article/**Supplementary Material**.

## Ethics statement

The animal study was reviewed and approved by Animal Experimentation Ethics Committee of the School of Medicine, Shanghai Jiao Tong University.

## Author contributions

ML and HM performed data processing, animal experiments as well as statistical analysis, and were major contributors to writing the manuscript. SZ conducted the experiments and YP took part in the statistical analysis. LD helped in the data collection. YZ revised the manuscript. PM conceived and designed this study and revised the manuscript. All the authors read and approved the final manuscript.
