# Supplementary material for: Potential therapeutic effect of NK1R antagonist in diabetic non-healing wound and depression
Source: Front Endocrinol (Lausanne). 2023 Jan 4;13:1077514. doi: 10.3389/fendo.2022.1077514 (PMC9845920; doi:10.3389/fendo.2022.1077514)
Supplement: Supplementary file 1 [file Table_1.docx]

**Table S1.** Blood glucose level of mice.

| **No.** | **Blood glucose level (mM)** | | | | | **Mean** |
| --- | --- | --- | --- | --- | --- | --- |
|  | Day -5 | Day -4 | Day -3 | Day -2 | Day -1 |  |
| 1 | 20.2 | 20.5 | 20.4 | 19.9 | 18.7 | 19.9 |
| 2 | 21.2 | 22.5 | 22.0 | 21.9 | 21.7 | 21.9 |
| 3 | 17.3 | 17.5 | 17.6 | 18.8 | 17.2 | 17.7 |
| 4 | 16.9 | 17.9 | 17.5 | 17.3 | 17.7 | 17.5 |
| 5 | 18.5 | 19.0 | 18.9 | 18.3 | 18.2 | 18.6 |
| 6 | 18.9 | 18.2 | 18.9 | 19.0 | 19.5 | 18.9 |
| 7 | 20.3 | 20.6 | 21.1 | 20.1 | 21.1 | 20.6 |
| 8 | 20.9 | 20.1 | 19.9 | 20.4 | 21.3 | 20.5 |
| 9 | 21.1 | 21.4 | 21.3 | 20.2 | 19.7 | 20.7 |
| 10 | 20.2 | 21.6 | 22.1 | 20.5 | 20.6 | 21.0 |
| 11 | 23.6 | 23.1 | 22.8 | 23.9 | 23.0 | 23.3 |
| 12 | 21.3 | 21.3 | 22.7 | 22.9 | 23.0 | 22.2 |
| 13 | 20.1 | 21.1 | 21.1 | 21.8 | 21.6 | 21.1 |
| 14 | 20.7 | 20.3 | 20.9 | 21.6 | 21.4 | 21.0 |
| 15 | 22.5 | 22.1 | 20.7 | 20.5 | 20.4 | 21.2 |
| 16 | 19.6 | 18.7 | 18.5 | 19.0 | 19.1 | 19.0 |
| 17 | 19.6 | 19.3 | 19.4 | 20.0 | 20.3 | 19.7 |
| 18 | 20.2 | 21.2 | 21.1 | 20.6 | 20.5 | 20.7 |
| 19 | 16.9 | 17.7 | 18.9 | 17.9 | 18.5 | 18.0 |
| 20 | 19.3 | 18.3 | 18.4 | 17.9 | 19.0 | 18.6 |
| 21 | 17.2 | 17.5 | 17.9 | 19.0 | 19.2 | 18.2 |
| 22 | 19.2 | 18.8 | 17.9 | 18.2 | 18.5 | 18.5 |
| 23 | 23.0 | 21.1 | 21.5 | 21.4 | 21.8 | 21.8 |
| 24 | 20.2 | 21.6 | 20.6 | 20.4 | 23.4 | 21.2 |
