# Supplementary material for: Potential therapeutic effect of NK1R antagonist in diabetic non-healing wound and depression
Source: Front Endocrinol (Lausanne). 2023 Jan 4;13:1077514. doi: 10.3389/fendo.2022.1077514 (PMC9845920; doi:10.3389/fendo.2022.1077514)
Supplement: Supplementary file 2 [file Table_2.docx]

**Table S2**. The primer sequences for qRT-PCR.

| **Primers** |  |
| --- | --- |
| mouse GAPDH F | 5' CCC GTA GAC AAA ATG GTG AA 3' |
| mouse GAPDH R | 5' TGC CGT GAG TGG AGT CAT AC 3' |
| mouse GAD1 F | 5' TTC TTG CTG GAA GTG GTA GAC 3' |
| mouse GAD1 R | 5' GTG TGG GTG GTG GAA ATC 3' |
| mouse ALDH1B1 F | 5' CGG GCT CCA CTG AGG TAG 3' |
| mouse ALDH1B1 R | 5' GCT CCA GGG TGA CTC TCT TG 3' |
| mouse Foxp2 F | 5' GCA GGT GGT ACA GCA GTT AGA 3' |
| mouse Foxp2 R | 5' GTG GTA GGG GTT TGA GGT AAG 3' |
