# Supplementary material for: Potential therapeutic effect of NK1R antagonist in diabetic non-healing wound and depression
Source: Front Endocrinol (Lausanne). 2023 Jan 4;13:1077514. doi: 10.3389/fendo.2022.1077514 (PMC9845920; doi:10.3389/fendo.2022.1077514)
Supplement: Supplementary file 3 [file Table_3.docx]

**Table S3.** List of antibodies.

| **Primary antibodies** |  |
| --- | --- |
| β-Actin | Proteintech, 66009-I-Ig, 42 KDa, mouse, 1:2000 |
| FOXP2 | Proteintech，20529-1-AP，70-80KDa，rabbit, 1:1000 |
| GAD1 | Proteintech，10408-1-AP，67KDa，rabbit, 1:1000 |
| ALDH1B1 | Proteintech，15560-1-AP，55/57KDa，rabbit, 1:1000 |
| **Secondary antibodies** |  |
| goat anti-mouse IgG | Abcam, ab205719, goat, 1:5000 |
| goat anti-rabbit IgG | Abcam, ab6721, goat, 1:5000 |
